# Drilling Performance Evaluation of Additively Manufactured Continuous Carbon Fiber Reinforced Thermoplastic Composites

**DOI:** 10.3390/polym18040544

**Published:** 2026-02-23

**Authors:** Altuğ Uşun, Cem Alparslan, Muhammed Furkan Erhan, Hamdi Kuleyin, Recep Gümrük, Şenol Bayraktar

**Affiliations:** 1 Medical Device Design and Production Application and Research Center, Karadeniz Technical University, 61080 Trabzon, Türkiye; altug@ktu.edu.tr (A.U.); rgumruk@ktu.edu.tr (R.G.); 2 HPET Engineering, 61081 Trabzon, Türkiye; hamdi.kuleyin@erdogan.edu.tr; 3 Department of Mechanical Engineering, Faculty of Engineering and Architecture, Recep Tayyip Erdoğan University, 53100 Rize, Türkiye; senol.bayraktar@erdogan.edu.tr; 4 Department of Manufacturing Engineering, Faculty of Technology, Gazi University, 06560 Ankara, Türkiye; furkanerhan@gazi.edu.tr

**Keywords:** Continuous Fiber-Reinforced Thermoplastic Composites (CFRTPs), material extrusion, additive manufacturing, drilling, delamination

## Abstract

This study investigates the machinability of Continuous Fiber-Reinforced Thermoplastic Composite (CFRTP) produced via Material Extrusion (MEX) additive manufacturing, focusing on drilling as a critical post-processing step in hybrid manufacturing. CFRTP components, fabricated from 3K carbon fibers and a PLA matrix, were subjected to systematic drilling tests under varying cutting speeds (50–110 m/min) and feed rates (0.06–0.24 mm/rev). Thrust force (Fz) and torque (Mz) were recorded using a high-precision dynamometer to evaluate the influence of cutting parameters on mechanical loads and damage mechanisms. Results indicate that increasing the feed rate significantly increases Fz and Mz, promoting fiber pull-out, delamination, and edge deformation, particularly at hole entry and exit regions. Conversely, higher cutting speeds reduce Fz and Mz due to thermal softening of the PLA matrix, enabling more controlled fiber–matrix interaction. Microscopic analyses revealed that damage severity correlates strongly with mechanical load levels. While high feed rates caused pronounced surface irregularities and matrix smearing, low feed rates combined with high cutting speeds yielded smoother hole morphology and preserved fiber–matrix integrity. The study concludes that optimal drilling conditions for CFRTP materials involve low feed rates and high cutting speeds, minimizing mechanical loads and suppressing damage formation. These findings provide a scientific basis for precision finishing strategies in hybrid manufacturing, enhancing dimensional accuracy and structural reliability of CFRTP components for advanced engineering applications.

## 1. Introduction

Continuous Fiber-Reinforced Thermoplastic Composites (CFRTPs) printing is a relatively new additive manufacturing method with growing interest in fields such as aerospace, automotive, marine, and structural engineering [1,2]. The integration of continuous reinforcing fibers within thermoplastic matrices during the additive manufacturing process offers opportunities to produce lightweight, high-strength components with tailored mechanical properties that can achieve performance comparable to or exceeding that of traditional manufacturing methods [3,4,5]. This capability positions CFRTP printing as a critical technology for advancing next-generation engineering solutions where the performance-to-weight ratio and design flexibility are important [6,7].

Material Extrusion (MEX) is one of the most widely studied methods for fabricating CFRTP parts, with several techniques developed to address the challenges of integrating continuous fibers [8,9]. Current techniques include in situ fiber impregnation systems [10,11,12], pre-impregnated filament extrusion [13,14,15], dual-nozzle configurations for simultaneous matrix and fiber deposition [16,17,18], and specialized heating mechanisms to ensure optimal fiber–matrix adhesion [19,20]. These diverse approaches reflect the ongoing evolution of CFRTP printing technologies. Researchers and manufacturers seek to optimize processing parameters, material compatibility, and structural performance outcomes [21,22,23].

However, despite recent progress, CFRTP printing still faces significant challenges that affect part quality and reliability. Common issues include fiber breakage, uneven fiber placement, thermal degradation, poor interlayer adhesion, and the complex relationship between process parameters and mechanical properties [24,25]. These problems can result in parts with lower structural integrity, dimensional errors, and inconsistent performance—limiting the broader use of CFRTP printing in critical applications.

To address these limitations, many researchers have focused on developing advanced printing path strategies to improve fiber orientation and reduce stress concentration. These include topology optimization, stress-based fiber placement, continuous fiber path planning, and multi-objective algorithms that consider both mechanical and manufacturing factors [26,27]. For example, Zha et al. [28] proposed a machine learning-assisted toolpath planning method for the additive manufacturing of CFRTP. Their approach uses a convolutional neural network enhanced with a self-attention mechanism to predict regular principal stress direction fields, enabling the generation of stress-aligned fiber paths. The extracted toolpaths are further processed for manufacturability by applying continuity optimization, curvature correction, and spacing evaluation. This method significantly improves computational efficiency while maintaining mechanical performance and manufacturability across various structural geometries.

While these methods have improved theoretical performance, practical challenges still prevent consistent real-world results and constrain the achievement of optimal manufacturing outcomes. To bridge the gap between design potential and actual performance, hybrid manufacturing combining additive and subtractive processes has been proposed [29,30,31]. Post-processing steps like machining can improve dimensional accuracy, surface finish, and part complexity beyond what additive methods alone can achieve. This combination of techniques offers a more flexible and precise manufacturing route. Drilling, in particular, stands out as one of the most critical machining processes in additively manufactured CFRTP. The thrust force (Fz) and torque (Mz) generated during drilling not only determine machinability but can also seriously compromise the structural integrity of the final product by directly triggering damage mechanisms such as fiber breakage, delamination, heat accumulation, and matrix degradation [32,33,34]. In this context, to maintain the dimensional accuracy and service performance of CFRTP parts, it is essential that drilling be performed with optimal cutting parameters. However, systematic and experimental data on the drilling behavior of additively manufactured CFRTP materials with high fiber volume fractions are extremely limited in the literature. Therefore, a detailed study of advanced mechanical responses (i.e., Fz and Mz behavior) during drilling provides the scientific basis for the industrial applicability of the hybrid manufacturing concept and significantly contributes to the understanding of complex fiber–matrix interactions.

This study aims to experimentally investigate the mechanical response of CFRTP parts produced using additive manufacturing. In this context, Fz and Mz values measured under different drilling parameters were analyzed to assess the effects of cutting conditions on material integrity and machinability. Based on the findings, optimal drilling conditions were determined, providing a scientific basis for improving critical performance criteria such as dimensional accuracy, surface quality, and structural reliability of CFRTP components in hybrid manufacturing. Thus, the study aims to contribute to the development of design and manufacturing strategies for the high-precision finishing requirements of CFRTPs, thereby increasing their potential use in advanced engineering applications. The novelty of this work lies in the systematic investigation of drilling behavior in high fiber volume fraction CFRTP components produced via material extrusion, through the integrated use of force–torque measurements, response surface methodology (RSM), and detailed damage characterization within a unified experimental framework. This approach provides new insight into the coupled relationship between drilling parameters, mechanical load evolution, and damage mechanisms, which remains limited in the current literature.

## 2. Materials and Methods

### 2.1. Materials

This work employed 3K continuous carbon fiber (DowAksa, Istanbul, Turkey) as the reinforcement material, selected for its high tensile strength performance and stiffness. The fibers feature a nominal diameter of 7 µm, with mechanical properties including a tensile strength near 4900 MPa and an elasticity modulus of 245 GPa. As the thermoplastic matrix, polylactic acid (PLA) filament with a diameter of 1.75 mm was sourced from Porima. The PLA exhibits a tensile strength of 54.3 MPa and a modulus of elasticity of 2300 MPa, making it suitable for composite fabrication with high interfacial compatibility.

### 2.2. Manufacturing of the CFRTP Filaments

**Figure 1:**
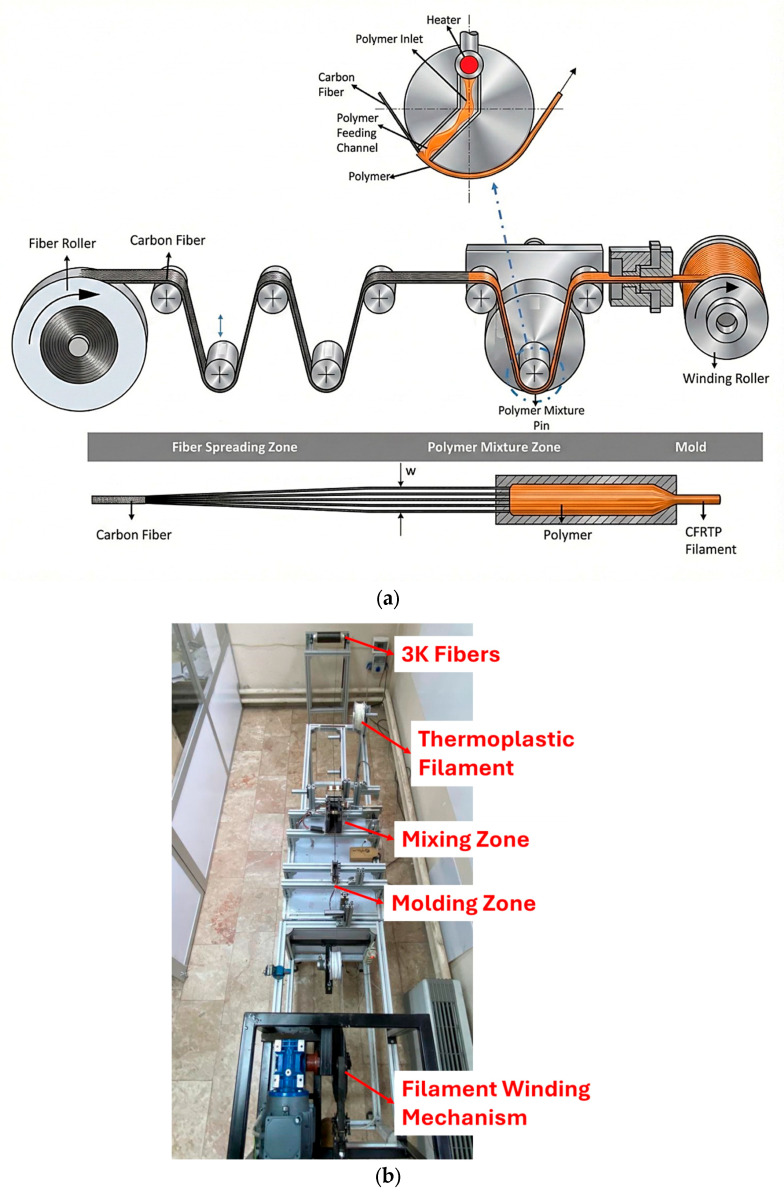

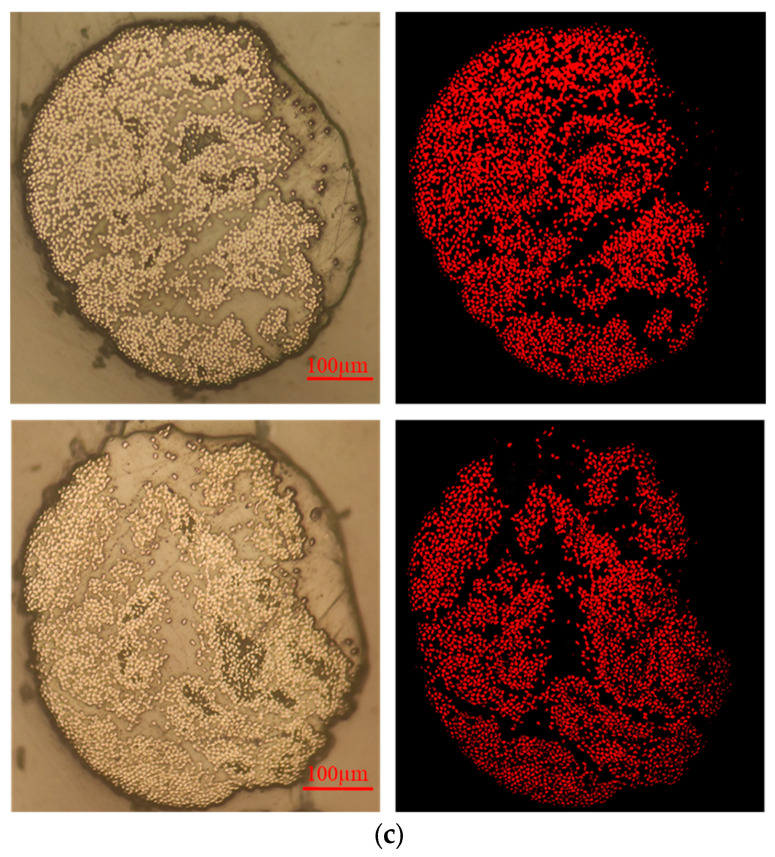
CFRTP PLA filament production line; (**a**) schematic image and (**b**) photograph of the manufacturing line; (**c**) optical micrographs of the filament cross-section and ImageJ-based area fraction.

Composite filaments were fabricated using a custom-built pultrusion-based system schematically given in Figure 1a, previously described in detail in related work [20]. This filament manufacturing system uses a continuous impregnation of 3K carbon fibers with a thermoplastic polymer by maintaining controlled temperature and tension parameters throughout the process. The produced CFRTP filaments consistently exhibit a fiber volume fraction close to 50% (Figure 1c) and an average diameter of about 0.55 mm [5]. 

The fiber volume fraction (V_f_) of the produced CFRTP filaments was consistently maintained at approximately 50%, a value that represents a high-performance for continuous fiber additive manufacturing. Optical cross-sectional images of the full filament diameter have been examined, and the fiber volume fraction was quantified using ImageJ-based area fraction analysis (Figure 1c). While foundational work by Matsuzaki et al. [12] estimated the practical upper limit for in-nozzle impregnation to be approximately 40-50% due to the physical constraints of the nozzle, subsequent advancements in pre-impregnation techniques have allowed for higher concentrations. In our previous research, V_f_ levels reached as high as 58%; however, these higher fractions introduced significant challenges in printability and consistent extrusion [20]. Thus, the 50% V_f_ used in this study was selected as the optimal balance for high-strength requirements and reliable processing [5].

This fiber content is well-documented in recent high-performance studies. Parker et al. [35,36] successfully utilized 50% V_f_ CCF/PPS filaments to set new benchmarks in additive manufacturing strength. Further pushing these limits, Zhang et al. [37] achieved a 54% V_f_ with PA6/carbon fiber, and Pichard et al. [38] reached 55% V_f_ using PA12/basalt fibers with successful printing and low porosity. In specialized applications, even higher values have been reported, such as 60% V_f_ tapes and 65% V_f_ prepreg filaments [39,40].

The setup consists of four main sections (Figure 1a,b): a fiber spreading unit, a polymer impregnation zone, a molding section, and a filament winding zone, following a melt-impregnation approach similar to a pultrusion process [20]. In the fiber spreading section, continuous 3K carbon fiber tows are guided over curved rollers that apply controlled lateral tension, causing the fiber bundle to open and spread. This spreading significantly increases the effective fiber–matrix contact area, shortens the impregnation path length, and promotes more efficient polymer penetration, which is critical for achieving homogeneous wetting and high mechanical performance. In the polymer impregnation zone, the thermoplastic filament is heated and molten using cartridge-heated rollers, and the melt is distributed onto the spread fibers through a specially designed impregnation roller featuring a shallow radial channel. This geometry enables the molten polymer to be forced uniformly across the fiber bundle. Additional rollers apply pressure and shear to further homogenize the fiber–matrix mixture and minimize resin-rich regions. Following impregnation, the composite exits the impregnation zone as a thin, fiber-rich strip and enters the molding section, where it is gradually reshaped into a circular filament. To avoid fiber damage and flow instabilities, shaping is performed in multiple steps using freely rotating forming rollers with semicircular grooves, followed by a conical shaping element that ensures dimensional stability and preserves fiber alignment. A final interchangeable mold and nozzle define the filament diameter and enable control of the fiber volume fraction. In the final stage, the filament is continuously drawn and collected using a dual-motor winding system, consisting of a haul-off motor that controls pulling force and impregnation time, and a low-torque winding motor that ensures stable filament collection without introducing excessive tensile stresses. This configuration allows continuous production of CFRTP filaments with stable geometry, controlled fiber volume fraction, and suitability for material extrusion–based additive manufacturing.

This process allows for continuous and consistent production of CFRTP filaments with good fiber alignment and impregnation quality, both of which are essential for achieving high mechanical performance in printed parts.

### 2.3. Additive Manufacturing of CFRTP Samples

Machinability test samples were fabricated using a Creality CR-6 SE fused filament fabrication (MEX) 3D printer (Shenzhen Creality 3D Technology Co., Ltd., Shenzhen, China). The printing was performed using the pre-impregnated CFRTP filaments described previously. A nozzle temperature of 230 °C and a bed temperature of 60 °C were used, with a layer height of 0.2 mm and a print speed of 5 mm/s. These parameters were selected to ensure consistent filament deposition, proper fiber alignment, and sufficient interlayer bonding suitable for subsequent machining tests.

To enable stable printing at high fiber volume fractions, a custom nozzle with an integrated compaction function was employed [41]. The nozzle applies additional pressure during deposition, promoting inter-filament consolidation and reducing interlayer voids. As a result, the printed CFRTP parts exhibit significantly reduced visible layer lines, leading to a microstructure that may visually resemble compression-molded composites, despite being produced via filament extrusion.

**Figure 2:**
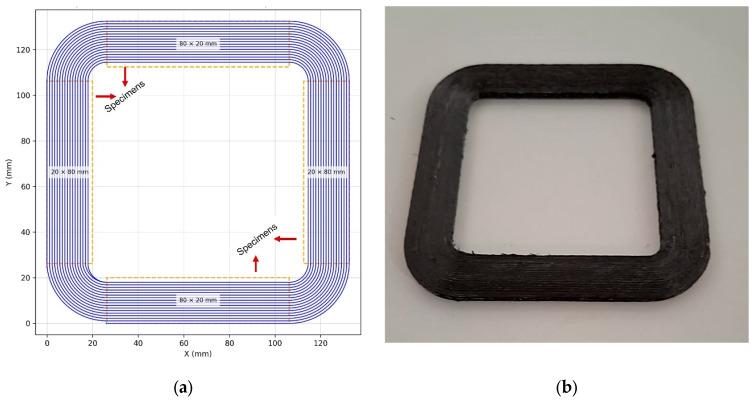
Material production: (**a**) Printing path of the continuous fiber part and (**b**) printed part image.

To eliminate the need for in-process cutting of continuous fibers, a custom Python-based scripting routine was employed to generate a fully continuous g-code for four rectangular specimens. The resulting toolpath, shown in Figure 2a, was deliberately designed with large-radius curvature transitions to minimize fiber buckling and ensure stable fiber–matrix co-deposition. This methodical toolpath planning was essential for preserving continuous fiber alignment throughout the printing process and for maintaining interlayer mechanical continuity. Also, the drilling test specimens were obtained by precision cutting with diamond cutting tools from the regions indicated on the printing path illustrated in Figure 2a. The part printed using CFRTP PLA filament via g-code–controlled fused filament fabrication is shown in Figure 2b. 

### 2.4. Mechanical Test

To obtain the mechanical properties and interlayer bonding quality of CFRTP PLA specimens, interlaminar shear strength (ILSS) tests were performed. ILSS specimens were printed with the same printing parameters as determined below in dimensions of 40 mm × 16 mm × 3.4 mm. Mechanical tests were performed with an Instron-3382 Universal testing machine at a crosshead velocity of 0.01667 mm/s. In tests, the supports were positioned with a 32 mm distance between the points of contact. Also, at least three ILSS tests were performed to ensure the test repeatability. 

### 2.5. Drilling Test

**Figure 3:**
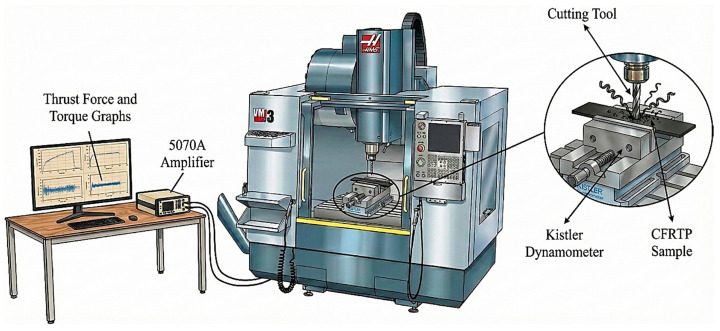
Drilling experiment setup.

Extensive drilling tests were conducted to determine the machinability characteristics of MEX manufactured CFRTP components. The experimental studies were carried out on a CNC vertical machining center (HAAS VF-2SS, 22.4 kW, Haas Automation, Inc., Oxnard, CA, USA) offering high rigidity and dynamic stability. The magnitudes of the Fz and Mz generated during the drilling process were recorded with high precision using a Kistler dynamometer; signal processing was performed via a Kistler 5070A amplifier module (Kistler Instrumente AG, Winterthur, Switzerland) to ensure data integrity. The measurements were transferred to a digital environment via a real-time data acquisition interface (Figure 3).

An Ø6 mm uncoated carbide drill bit was used as the cutting tool; all experiments were conducted under uncooled (dry) cutting conditions to evaluate the thermomechanical effects related to fiber–matrix interaction in a straightforward manner. The specimens were aligned with the tool axis, and the vibration damping characteristics of the clamping system were improved, minimizing the reflection of micro-deviations on the during cutting in the measurements. Each drilling operation was performed with a constant cutting depth of 7 mm. Three different cutting speeds (50, 70, 90, and 110 m/min) and three different feed rates (0.06, 0.12, 0.18, and 0.24 mm/rev) were selected to investigate the effects of cutting parameters on machining loads. The selected ranges of cutting speed and feed rate were determined based on literature recommendations for drilling fiber-reinforced polymer composites, preliminary experimental trials, and the operational limits of the machine–tool–material system. These ranges were chosen to represent a stable machining window that avoids excessive tool wear and catastrophic delamination while still allowing clear observation of variations in thrust force, torque, and damage evolution. Furthermore, the selected parameter domain provides a sufficiently wide experimental space to enable reliable modeling of linear, nonlinear, and interaction effects within the response surface methodology framework.

At least four independent repetitions were performed for each parameter combination; thus, statistical reliability was ensured. Damage types that may occur after drilling, such as fiber detachment, delamination, entry-exit edge deformations, and matrix cracks, were evaluated by correlating them with measured force and moment characteristics. In this way, causal connections between shear mechanics and fiber–matrix detachment behavior were revealed through a multidimensional analysis approach. In addition, machined surfaces and hole cross-sections obtained after the drilling process were examined under an optical microscope; microstructural damage mechanisms such as fiber fracture, fiber detachment, matrix cracks, and delamination were visualized through high-resolution cross-sectional images. The obtained microscopic findings were compared with the measured Fz and Mz signals, qualitatively and quantitatively confirming the relationship between mechanical loads and damage formation.

### 2.6. Response Surface Methodology (RSM) Analysis 

Response Surface Methodology (RSM) was used to model and optimize the Fz and Mz generated during the drilling process of continuous carbon fiber reinforced thermoplastic composites, depending on the process parameters. RSM analysis is a powerful method that allows experimental design, statistical modeling, and the determination of interactions between process parameters. Statistical analysis and mathematical modeling of experimental data were performed using Minitab 19.0 (Minitab Inc., State College, PA, USA) software. V and f were selected as independent variables in the analysis; Fz and Mz, measured during the drilling process, were considered as response variables. A quadratic regression model was constructed to reveal both linear and nonlinear effects, as well as interaction effects, of the selected parameters.

The statistical significance of the developed RSM model was evaluated using analysis of variance (ANOVA) [42]. The adequacy and reliability of the model were analyzed using the coefficient of determination (R^2^), adjusted coefficient of determination (Adj-R^2^), estimated coefficient of determination (Pred-R^2^), F-test, and p-values. In addition, residual analyses, normal probability plots, residual-predicted value plots, and histograms were examined to verify the model’s fit and validity with experimental data.

A standardized effects Pareto chart was created to determine the relative effects of the factors and interaction terms included in the model on the response. Furthermore, contour and three-dimensional response surface (3D surface) plots were used to visually illustrate the combined effects of V and f on Fz and Mz. The resulting statistical model was used as the basis for determining the appropriate drilling parameters that provide minimum Fz and Mz.

## 3. Results and Discussion

### 3.1. Mechanical and Microstructural Investigation of CFRTP Samples

**Figure 4:**
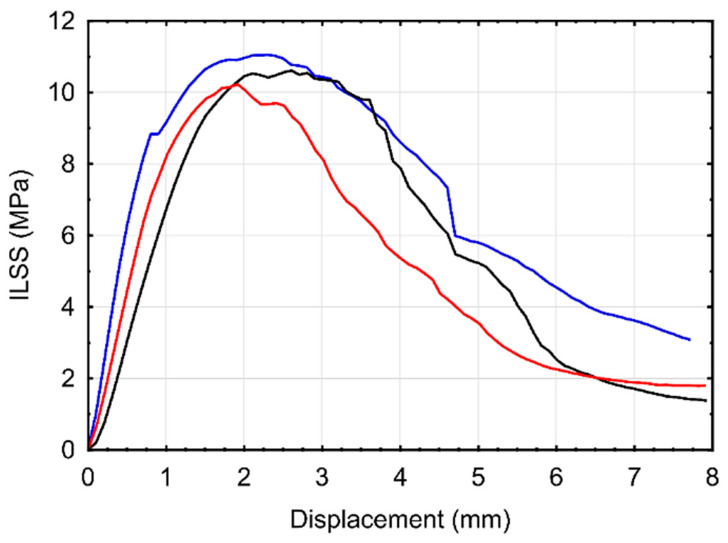
Interlaminar shear stress–displacement curves of samples obtained from ILSS tests.

The interlaminar shear performance of the additively manufactured CFRTP specimens was evaluated using short-beam shear (ILSS) tests to assess the quality of interlayer bonding inherent to the printing process. Representative ILSS-displacement responses are shown in Figure 4. The specimens exhibited an average ILSS of 10.63 MPa with a standard deviation of 0.34 MPa, indicating moderate interlaminar load-bearing capacity.

All specimens demonstrated a characteristic nonlinear shear response, comprising an initial steep increase in shear stress, followed by a distinct peak and a gradual post-peak softening regime. Such behavior is typical of CFRTP produced via MEX, where interlaminar failure is governed by a combination of matrix yielding, interfacial debonding, and progressive delamination rather than abrupt brittle fracture [43,44]. Similar failure characteristics have been reported in systems with limited fiber–matrix interfacial bonding and incomplete interlayer consolidation. Additionally, this response can also be attributed to the layer-by-layer deposition mechanism inherent to the additive manufacturing process, which promotes the formation of interlaminar voids and restricts polymer chain diffusion across adjacent layers, especially weak in CFRTP printing [45,46,47]. Accordingly, the ILSS–displacement curves exhibit an early stress drop following the peak load, suggesting premature initiation of interlaminar cracking and unstable shear damage propagation.

**Figure 5:**
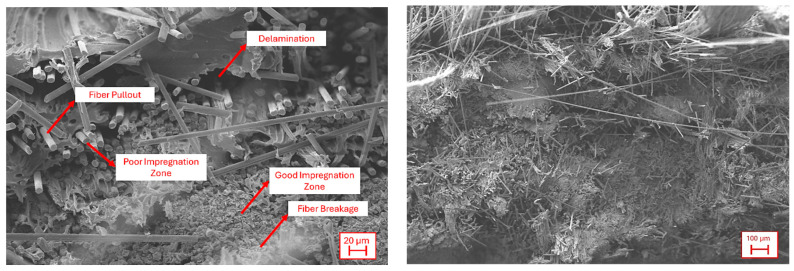
SEM images of the failed sample surface.

Overall, the ILSS values obtained in this study fall within the range commonly reported for additively manufactured continuous CF/PLA systems, where residual porosity, fiber turning points, and layer discontinuities constrain interlaminar performance [44,48]. The measured ILSS level therefore represents a realistic baseline for as-printed CFRTP components and provides a relevant mechanical context for interpreting the drilling-induced damage and machinability behavior discussed in subsequent sections. To further elucidate the interlaminar shear behavior, the fracture surfaces of ILSS-tested specimens were examined using scanning electron microscopy (SEM). Representative SEM images are presented in Figure 5, highlighting the dominant damage mechanisms governing interlaminar failure in the as-printed CFRTP structures. The fracture surfaces are characterized by extensive fiber pull-out, interlaminar separation, and localized delamination, which directly explain the moderate ILSS values measured in this study. In large regions of the fracture surface, carbon fibers are observed to be cleanly extracted from the matrix, leaving behind smooth fiber imprints and voided matrix regions.

In addition to fiber pull-out, interlaminar delamination planes are clearly visible, confirming that crack propagation preferentially occurs along weak interlayer interfaces formed during the layer-by-layer deposition process. These delaminated regions correlate well with the early stress drop observed in the ILSS–displacement curves, indicating unstable shear damage initiation once the interlaminar interface reaches its critical stress level. 

Despite the dominance of pull-out-driven failure, SEM observations also reveal localized zones of excellent fiber impregnation, where the polymer matrix tightly encapsulates the carbon fibers with minimal interfacial gaps. In these regions, fracture occurs predominantly through fiber breakage rather than pull-out, suggesting efficient load transfer from the matrix to the fibers. The coexistence of these two distinct failure modes, fiber pull-out in poorly impregnated regions and fiber breakage in well-impregnated zones, highlights the heterogeneous nature of interlayer consolidation in additively printed CFRTP structures. While the overall ILSS is governed by the weakest interfacial regions, the presence of locally well-bonded zones demonstrates that the filament impregnation quality and fiber–matrix compatibility are intrinsically sufficient, but spatially inconsistent across the printed part. This heterogeneous interlaminar structure is particularly relevant for subsequent machining operations, as regions dominated by fiber pull-out and weak interfaces are more susceptible to drilling-induced delamination and fiber tearing, whereas well-impregnated zones are expected to exhibit more stable cutting behavior and reduced damage evolution.

### 3.2. Fz and Mz Characteristics during Drilling of CFRTP Samples

The mechanical responses of CFRTP specimens produced by the MEX method during drilling were evaluated based on Fz and Mz values measured under different cutting speeds and feed parameters. The results show that cutting parameters have a significant and systematic effect on both Fz and Mz.

**Figure 6:**
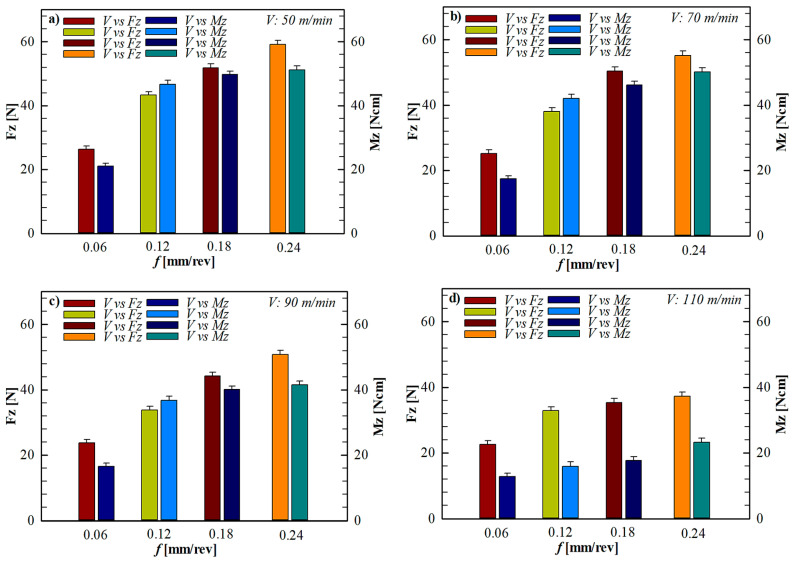
Effect of feed rate on Fz and Mz at different constant cutting speed: (**a**) 50 m/min, (**b**) 70 m/min, (**c**) 90 m/min, and (**d**) 110 m/min.

Experimental data revealed that the Fz and Mz values increased consistently at all cutting speeds with increasing feed rate. The effect of feed rate under constant cutting speeds is presented in Figure 6. As seen in Figure 6, increasing the feed rate from 0.06 mm/rev to 0.24 mm/rev at all cutting speeds of 50, 70, 90, and 110 m/min resulted in a significant increase in Fz and Mz values. For example, at a cutting speed of 50 m/min, increasing the feed rate from 0.06 mm/rev to 0.24 mm/rev increased the Fz from approximately 26.3 N to 59.08 N, and the Mz value increased from 21.01 to 51.20 N. A similar trend was observed at cutting speeds of 70, 90, and 110 m/min. This can be attributed to the fact that with increasing feed rates, the cutting tool has to remove more material per unit time, and to the increased mechanical resistance on the continuous fiber bundles [49]. Forcing continuous fibers to be partially pulled rather than cut leads to force concentrations in regions not adequately supported by the matrix. This behavior is consistent with previous studies on the puncture of CFRTP [2,34].

**Figure 7:**
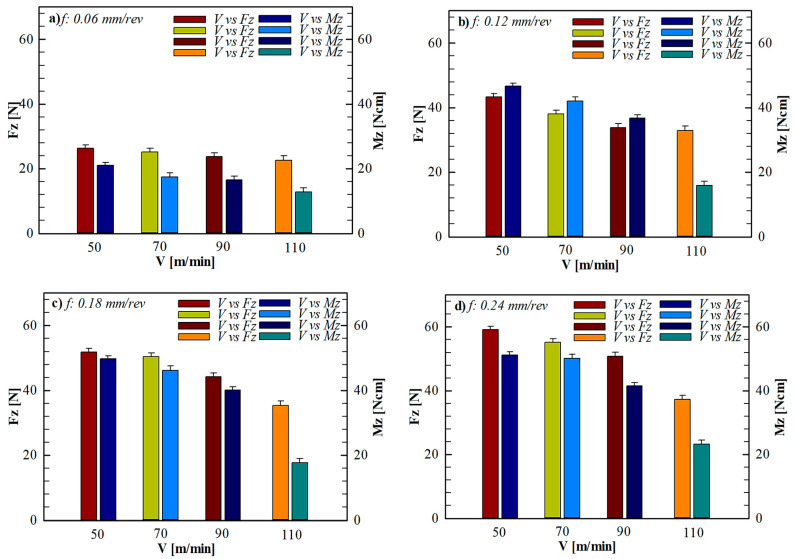
Effect of cutting speed on Fz and Mz at different constant feed rates: (**a**) 0.06 mm/rev, (**b**) 0.12 mm/rev, (**c**) 0.18 mm/rev, and (**d**) 0.24 mm/rev.

When the effect of cutting speed was examined, a general decrease in Fz and Mz values was observed with increasing cutting speed at the same feed rate. The effect of cutting speed under constant feed rates is shown in detail in Figure 7. As seen in Figure 7, increasing the cutting speed from 50 m/min to 110 m/min at all feed levels led to a gradual decrease in Fz and Mz values. Especially under the low feed condition (0.06 mm/rev), increasing the cutting speed from 50 m/min to 110 m/min reduced the Fz from 26.3 N to 22.61 N and the Mz value from 21.01 to 12.85. This decrease can be attributed to the fact that the temperature generated in the cutting zone due to the increased cutting speed softens the thermoplastic PLA matrix and reduces the shear resistance at the fiber–matrix interface. Although temperature was not directly measured in the present study, this interpretation is supported by well-established findings in the literature on thermoplastic composite machining. Thermal softening associated with increased shear rate has been reported to reduce mechanical loads in continuous fiber-reinforced thermoplastic composites [33].

When the Mz values are examined, it is seen that the Mz increases significantly with increasing feed rate, similar to the Fz. When Figure 6 and Figure 7 are evaluated together, it is understood that the high Mz values measured, especially at low cutting speeds and high feed rates indicate the formation of complex tool–fiber interactions depending on the fiber orientation around the hole. This finding reveals that the Mz signal is an important parameter reflecting not only cutting resistance but also fiber orientation and potential damage formation [34,50].

Overall, combinations of low feed rates and high cutting speeds appear to offer more stable drilling behavior for CFRTP materials. The lower Fz and Mz values measured under these conditions contribute to the suppression of damage mechanisms such as delamination, fiber shrinkage, and matrix cracks. The relationship between the measured mechanical loads and the types of damage observed at the hole entry, exit, and inner surface is examined in detail in the next section.

### 3.3. Drilling-Induced Damage Analysis at Hole Entrance, Exit and Inner Surfaces

**Figure 8:**
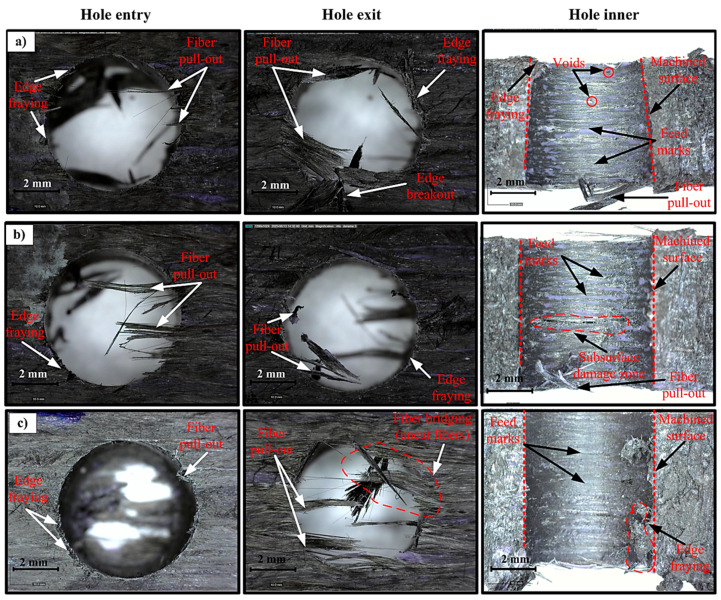

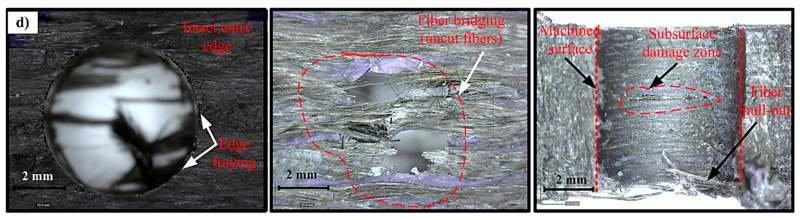
Optical microscope images showing drilling-induced damage in CFRTP samples under different cutting conditions: (**a**) 50 m/min–0.06 mm/rev, (**b**) 50 m/min–0.24 mm/rev, (**c**) 110 m/min–0.06 mm/rev, and (**d**) 110 m/min–0.24 mm/rev.

The types of damage that occur in CFRTP materials during drilling exhibit different morphological characteristics at the hole entry, hole exit, and hole interior surfaces, depending on the cutting parameters. Fiber shrinkage, delamination, matrix cracks, and irregular surface formations occurring in these regions of CFRTP materials are directly related to the penetration Fz and Mz levels. Therefore, regional assessment of post-drilling damage is critical for understanding the effects of mechanical loads on material behavior [50,51,52]. Representative morphologies of the hole entrance, exit, and inner surface are given comparatively in Figure 8 under different cutting conditions.

Investigations at the hole entry points show that significant surface deformations occur, especially at high feed rates and low cutting speeds (Figure 8b). Under these conditions, the sudden increase in Fz at the beginning of the drilling process causes the fibers to be partially pulled instead of cut, and highlights damage mechanisms such as edge fraying and fiber pull-out at the entry edge. It has been widely reported in the literature that such damage observed at the hole entry point in CFRTP is related to the cutting tool starting to cut the fibers without adequate support [51,53]. In contrast, under low feed rate and high cutting speed conditions, smoother entry edges and significantly reduced fiber pulling were observed (Figure 8c). This finding is consistent with the lower Fz values reported in Section 3.2.

Exit zones are among the most susceptible areas to damage in CFRTP materials. In the samples examined, fiber pulling/breakage at the exit edge, edge deformations, and exit-edge breakout (push-out type damage) were observed to be concentrated, especially at high feed rates (Figure 8b,d). The decrease in back support effect in the final stage of the drilling process and the approach of maximum Fz facilitate damage formation in this region. This mechanism is considered one of the main causes of delamination in drilling composite materials, and the Fz has been emphasized as a critical parameter in many studies [54,55]. It is also noteworthy that under certain conditions, the hole opening is partially closed by fibers, and fiber bridging (uncut fibers) behavior develops (Figure 8c,d). In contrast, at low feed rates and high cutting speeds, the damage in the exit zone remains more limited and the severity of the damage is significantly reduced (Figure 8c).

Microscopic examination of the hole interior surfaces and cross-sectional images reveals that damage is not limited to the entry and exit regions but can extend throughout the hole (Figure 8). At high feed rates, feed marks/striations, matrix smearing, and localized damage areas are observed on the interior surface (Figure 8b,d). This situation can be attributed to increased Mz values resulting from the intense interaction of the drill’s peripheral cutting edges with the fibers. Previous studies have also reported that the hole interior surface morphology in additively manufactured CFRTP is an important indicator reflecting the severity of tool–fiber interaction [50]. Under low feed rate and high cutting speed conditions, it was observed that the inner surfaces were more homogeneous and the fiber–matrix interface was largely preserved (Figure 8c).

In general, it is understood that the types of damage observed at the hole entry, exit, and inner surfaces are significantly related to changes in Fz and Mz values. While damage density increased under conditions where high mechanical loads were measured, more controlled drilling behavior with limited damage was exhibited at cutting parameters where lower Fz and Mz values were obtained. These findings reveal that cutting parameters are fundamental elements that determine not only machinability performance but also hole quality and structural integrity in drilling CFRTP materials. In summary, under high feed rate conditions, increasing Fz and Mz levels increase edge damage at the hole entry/exit and tool marks/striations and surface irregularities on the inner surface of the hole; while combinations of low feed rates and high cutting speeds presented a morphology that limited the severity of damage with lower loads (Figure 8).

### 3.4. Statistical Analysis of Fz

The developed second-order RSM model represented the variation of axial drilling force (Fz) with cutting parameters with high accuracy (R^2^ = 98.24%, Adj-R^2^ = 97.36%, Pred-R^2^ = 94.93%). The statistical significance of the model was confirmed by ANOVA results (F = 111.56, *p* < 0.001), and the low contribution of the error term to the total variance (1.76%) demonstrated the consistency of the experimental data (Table 1).

ANOVA and Pareto analysis results revealed that f was the most dominant parameter affecting Fz (72.49% contribution). This was followed by cutting speed (V) (18.12%) and V × f interaction (4.45%) (Table 2). Increasing the feed rate increased the amount of fiber encountered by the cutting tool per unit time, causing a sudden load transfer at the fiber–matrix interface and leading to a significant increase in axial force. The effect of cutting speed was more limited due to the presence of continuous fiber [56]. Analysis of the quadratic terms showed that the feed rate exhibited nonlinear behavior (f^2^, *p* < 0.01), while the quadratic effect of the cutting speed was not significant [42]. This can be attributed to the fact that fiber drift and interface separation become dominant at high f. The significant finding of the cutting speed–feed rate interaction (*p* < 0.01) indicates that the negative effect of the feed rate can be partially suppressed at high V.

**Figure 9:**
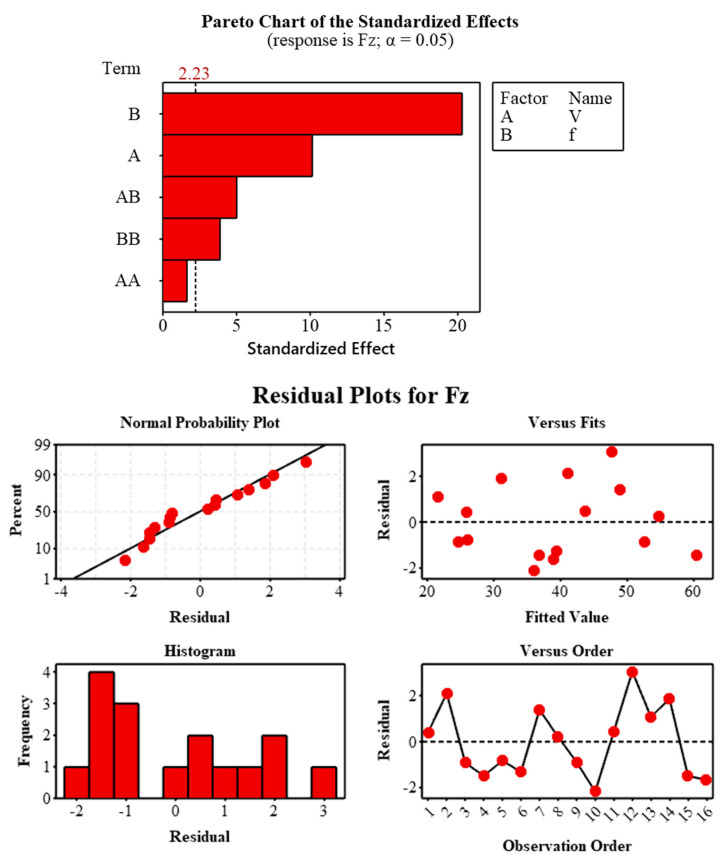
Pareto diagram of standardized effects and residual diagnostic plots for Fz (α = 0.05).

The analysis has now confirmed that the model assumptions are met and that the developed RSM model is reliable in terms of Fz estimation (Figure 9). In general, it has been determined that combinations of low feed rates and medium-high cutting speeds are more suitable in terms of limiting the axial force.

### 3.5. Statistical Analysis of Mz

The regression model developed using response surface methodology successfully represented the variation of drilling Mz with cutting parameters (R^2^ = 93.20%, Adj-R^2^ = 89.80%). The lower predicted R^2^ value (78.69%) indicates that torque behavior has a more complex structure compared to axial force. However, the ANOVA results confirmed that the model is statistically significant (F = 27.42, *p* < 0.001) (Table 3).

ANOVA and Pareto analysis revealed that V and f are the dominant parameters influencing Mz. Cutting speed contributed 39.78%, and feed rate contributed 37.25%; these two parameters had almost equal effects on Mz (Table 4). This indicates that torque is controlled by both peripheral cutting resistance and chip load. The significant value of the quadratic terms (V^2^ and f^2^, *p* < 0.05) indicates that Mz exhibits nonlinear behavior and that the torque increase accelerates, especially at high V and f. The V–f interaction was found to be borderline significant (p = 0.050), revealing that the combined variation of the parameters has a secondary but not negligible effect on Mz.

**Figure 10:**
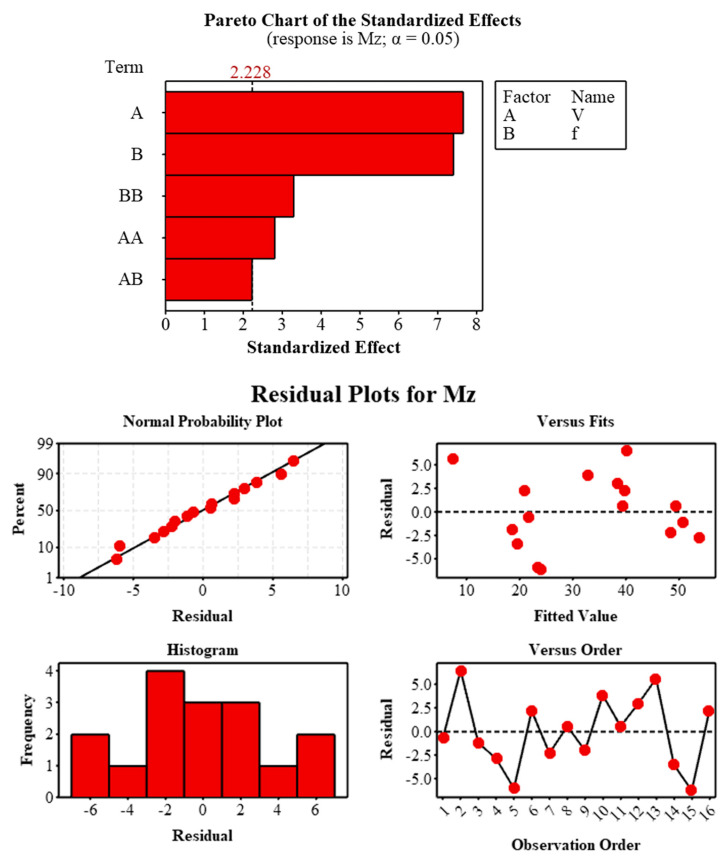
Pareto diagram of standardized effects and residual diagnostic plots for Mz (α = 0.05).

The analyses have now confirmed that the model assumptions are met and that the developed RSM model offers acceptable accuracy in terms of Mz estimation (Figure 10). The results show that medium V and low-to-medium f combinations are more suitable for torque control in drilling operations of continuous carbon fiber reinforced thermoplastic composites.

## 4. Conclusions

In this study, the machinability behavior of CFRTPss produced by MEX during drilling was investigated using a comprehensive experimental approach. The Fz and Mz signals generated during the drilling process were evaluated in relation to the cutting parameters; the obtained mechanical loads were interpreted together with the damage mechanisms observed in the hole entry and exit regions and on the inner surfaces. The main results obtained from the study are summarized below:

- Increasing the feed rate resulted in a significant and systematic increase in both Fz and Mz values at all cutting speeds. At high feed rates, mechanical resistance increased and drilling loads rose significantly due to the partial pulling of continuous fibers instead of cutting them.
- Increased mechanical loads have promoted the formation of fiber pulling, fiber breakage, edge distortions, and thrust-type damage, particularly in the entry and exit regions of the hole. This clearly demonstrates the damage-prone nature of drilling operations in CFRTP materials.
- Increasing the cutting speed resulted in a general decrease in Fz and Mz values under constant feed conditions. This trend is attributed to the thermal softening effect in the cutting zone, which reduces the shear strength of the thermoplastic matrix and leads to a more controlled cutting mechanism at the fiber–matrix interface.
- Combinations of low feed rates and high cutting speeds yielded the most stable drilling behavior; under these conditions, lower mechanical loads were measured, and the severity of damage at the hole entry and exit points and on the inner surfaces was significantly limited.
- Statistical analysis confirmed that the feed rate is the dominant parameter governing the axial Fz, whereas Mz is influenced by the combined and comparable effects of *V* and *f.* The developed quadratic regression models exhibited high statistical significance and predictive capability, validating the robustness of the experimental findings.
- The results further revealed significant nonlinear and interaction effects between *V* and *f*, particularly for Mz response. These interaction effects highlight the coupled nature of drilling mechanics in CFRTPs and demonstrate that unfavorable parameter combinations may amplify mechanical loads even when individual factors are kept within moderate ranges.
- Microstructural analyses have shown that under conditions where high Fz and Mz values are measured, significant penetration marks, matrix coating, and surface irregularities are concentrated on the inner surfaces of the holes; in contrast, fiber–matrix interface integrity is largely preserved at low load levels.
- The Mz signal has been identified as a critical indicator reflecting not only shear resistance but also fiber orientation and the complexity of tool-fiber interaction.

When these findings are considered together, it is concluded that low feed rates and high cutting speeds are the optimal machining regime for drilling CFRTP materials, minimizing mechanical loads and suppressing damage caused by drilling. This study provides a reliable scientific basis for high-precision finishing processes within hybrid manufacturing approaches for continuously fiber-reinforced composites produced by additive manufacturing. In this respect, the results obtained contribute to the wider and safer use of CFRTP components in industrial applications.

## Figures and Tables

**Table 1:** Model Summary.

S	R-sq	R-sq(adj)	PRESS	R-sq(pred)	AICc	BIC
1.91164	98.24%	97.36%	105.160	94.93%	86.62	78.03

**Table 2:** Analysis of Variance for Fz.

Source	DF	Seq SS	Contribution	Adj SS	Adj MS	f-Value	*p*-Value
Model	5	2038.34	98.24%	2038.34	407.67	111.56	0.000
Linear	2	1880.13	90.61%	1880.13	940.07	257.25	0.000
*V*	1	375.97	18.12%	375.97	375.97	102.88	0.000
*f*	1	1504.16	72.49%	1504.16	1504.16	411.61	0.000
Square	2	65.79	3.17%	65.79	32.89	9.00	0.006
V*V	1	10.10	0.49%	10.10	10.10	2.76	0.127
f*f	1	55.69	2.68%	55.69	55.69	15.24	0.003
2-Way Interaction	1	92.42	4.45%	92.42	92.42	25.29	0.001
V*f	1	92.42	4.45%	92.42	92.42	25.29	0.001
Error	10	36.54	1.76%	36.54	3.65		
Total	15	2074.88	100.00%				

**Table 3:** Model Summary.

S	R-sq	R-sq(adj)	PRESS	R-sq(pred)	AICc	BIC
4.63766	93.20%	89.80%	674.127	78.69%	114.98	106.39

**Table 4:** Analysis of Variance for Mz.

Source	DF	Seq SS	Contribution	Adj SS	Adj MS	f-Value	*p*-Value
Model	5	2949.1	93.20%	2949.1	589.81	27.42	0.000
Linear	2	2437.2	77.03%	2437.2	1218.61	56.66	0.000
*V*	1	1258.6	39.78%	1258.6	1258.57	58.52	0.000
*f*	1	1178.6	37.25%	1178.6	1178.65	54.80	0.000
Square	2	404.8	12.79%	404.8	202.40	9.41	0.005
V*V	1	170.6	5.39%	170.6	170.63	7.93	0.018
f*f	1	234.2	7.40%	234.2	234.17	10.89	0.008
2-Way Interaction	1	107.1	3.38%	107.1	107.05	4.98	0.050
V*f	1	107.1	3.38%	107.1	107.05	4.98	0.050
Error	10	215.1	6.80%	215.1	21.51		
Total	15	3164.1	100.00%				

## Data Availability

The original contributions presented in the study are included in the article. Further inquiries can be directed to the corresponding author.
